# Clinical effect of soft coagulation for air leak treatment during pulmonary lobectomy

**DOI:** 10.1186/s13019-022-01948-x

**Published:** 2022-10-04

**Authors:** Yuki Takahashi, Ryunosuke Maki, Kodai Tsuruta, Makoto Tada, Wataru Arai, Yuma Shindo, Yasuyuki Nakamura, Masahiro Miyajima, Atsushi Watanabe

**Affiliations:** Department of Thoracic Surgery, School of Medicine and Hospital, Sapporo Medical University, South 1, West 16, Chuo-ku, Sapporo, Hokkaido 060-8543 Japan

**Keywords:** Soft coagulation, VIO soft coagulation, Pulmonary resection, Pulmonary fistula, Macchiarini scale score

## Abstract

**Background:**

Soft coagulation using the VIO soft coagulation system is used to treat minor lung air leaks during pulmonary resection in Japan. We previously reported that it has a similar effect as the air leak treatment with fibrin glue. We evaluated the efficacy of soft coagulation using the VIO soft coagulation system for lung air leakage during pulmonary resection.

**Methods:**

Intraoperative air leaks from the interlobar lung parenchyma were observed in 42 of the 283 patients who underwent video-assisted thoracoscopic surgery lobectomy between 2016 and 2018. We retrospectively reviewed these 42 patients who were treated using the VIO soft coagulation system for air leaks. We classified the air leaks in to grades using the Macchiarini scale score and evaluated the surgical outcomes of air leak treatment.

**Results:**

Air leaks from the interlobar lung parenchyma having Macchiarini scale scores 1, 2, and 3 occurred in 8, 17, and 17 patients, respectively. In all the 8 patients with score 1 air leaks (100%), the air leaks could be controlled using the VIO soft coagulation system alone, and none had delayed pneumothorax requiring intervention. Of the score 2 and 3 air leaks, 52.9% and 35.3% were controlled using the VIO soft coagulation system alone, respectively.

**Conclusions:**

Macchiarini scale score 1 air leaks from the interlobar lung parenchyma could be well controlled using the VIO soft coagulation system. Therefore, soft coagulation with this system may be an alternative method for treating minor air leaks during pulmonary resection surgery.

## Background

Intraoperative air leaks reportedly occur in 44%–88% of patients undergoing lung resection [1, 2]. Currently, stapling, suturing, and sealing with surgical sealants are the treatment options for lung air leaks in thoracic surgery. The most popular surgical sealant for lung air leak treatment is fibrin glue [3–6]. In addition to artificial materials, the use of fibrin glue entails the risk of viral transmission because it is a plasma partition preparation [7]. Furthermore, fibrin glue is expensive.

The VIO soft coagulation system (VSCS) is a new device for tissue coagulation. The output voltage is automatically regulated without generating sparks or causing carbonization of tissues, thereby resulting in minimal damage to the surrounding tissues [8]. The VSCS is predominantly used to stop hemorrhage. Sakuragi et al. reported the achievement of hemostasis following pulmonary artery bleeding using a monopolar VSCS [9, 10]. Although this device is for hemostasis, some institutions in Japan have reported the efficacy of the VSCS for controlling lung air leaks [11, 12]. In a previous study in a mouse pulmonary air leak model, we reported that pressure resistance following soft coagulation with the VSCS was equivalent to that after sealing with fibrin glue [13]. Currently, in our institute, we use the VSCS for minor air leak treatment during pulmonary resection.

This study aimed to clarify the efficacy of the VSCS for lung air leak treatment. We determined the surgical outcomes of patients who underwent air leak treatment with the VSCS for interlobar lung parenchyma during pulmonary resection and the possible relationship between outcomes and the grade of air leak.

## Methods

Our institutional review board approved this retrospective study (approval number 322-310), and the requirement for obtaining informed consent was waived.

### Patients

**Fig. 1:**
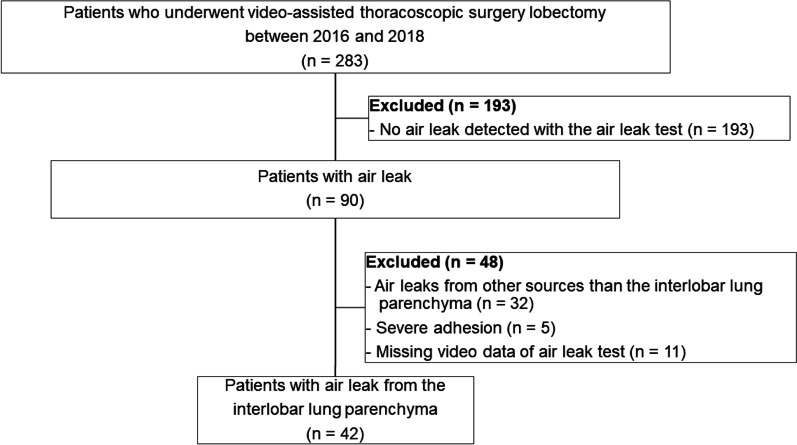
Flowchart illustrating the patient selection process

Medical records, including intraoperative videos of 283 patients, who underwent video-assisted thoracoscopic surgery (VATS) lobectomy for primary lung cancer between January 2016 and December 2018 at our institute, were reviewed. Patients with no air leak in the air leak test, severe pleural adhesion, air leak from lung injury other than to the interlobar lung parenchyma, or missing video data from the air leak test were excluded. Ultimately, 42 patients were included in this study (Fig. 1). Patients who visited our institution after 2019 were not included in this study. Robot-assisted thoracoscopic surgery was introduced in our institution at this time, and the procedure for air leak treatment was changed; for instance, suturing for air leak treatment was occasionally performed before soft coagulation.

### Surgical procedures

Patients underwent VATS lobectomy in the lateral position under general anesthesia with selective lung ventilation. Lung deflation was maintained throughout most of the operative period.

**Fig. 2:**
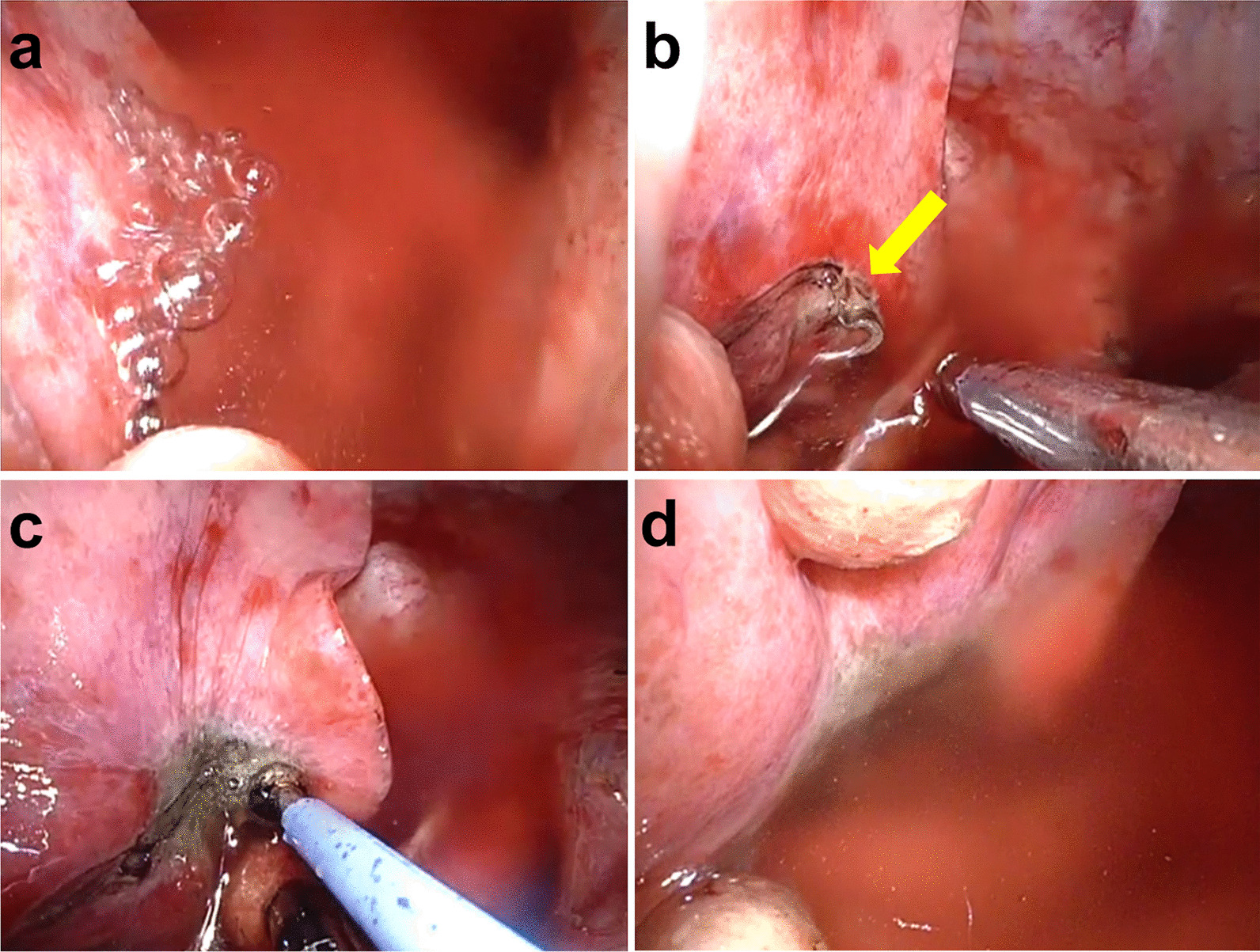
Thoracoscopic view: **a** an intraoperative air leak from the interlobar lung parenchyma; **b** the yellow arrow indicates pulmonary fistula; **c** soft coagulation with the VIO soft coagulation system set at 80 Watt/Effect 5 for air leak treatment; and **d** confirmation of air leak stoppage

After completing the lobectomy, an air leak test was performed before wound closure. The air leak test was confirmed by reinflation of the lung on the affected side at a maximum pressure of 15 cmH_2_O for 1–2 min after water sealing. Soft coagulation with the VSCS at 80 Watt/Effect 5 was performed when air leakage from the interlobar lung parenchyma was detected (Fig. 2). An additional procedure for pulmonary fistula was performed after the VSCS only when the air leak was uncontrolled. Finally, a chest tube was placed, and the wound was closed.

### Classification of air leak

Air leaks were graded using the Macchiarini scale score (MSS) after confirmation with surgical videos. The MSS air leaks were classified as 0 (absent, no apparent leak), 1 (mild, countable bubbles), 2 (moderate, stream of bubbles), and 3 (severe, coalesced bubbles) [2, 14]. Three thoracic surgeons independently classified the air leaks with the MSS using the same videos, and the air leaks were classified into grades that two or all three surgeons agreed upon.

### Statistical analysis

The chi-square test was utilized for the comparison of categorical variables. The significance level was set at a *p* value of 0.05. All statistical analyses were performed using SPSS software (version 22.0; IBM Corp., Armonk, NY, USA).

## Results

### Patient characteristics and air leak classification

Between January 2016 and December 2018, 42 of the 283 patients who were reviewed, experienced intraoperative air leaks from the interlobar lung parenchyma, as confirmed via an air leak test. The air leaks in 8, 17, and 17 patients were classified as MSS 1, 2, and 3, respectively (Table 1).

**Table 1:** Characteristics of patients with air leaks from the interlobar lung parenchyma during pulmonary lobectomy

	Number (%) or median [range] (N = 42)
Sex (male/female)	30/12
Age (years)	71.5 [56–86]
Pulmonary emphysema (+ / −)	20/22
COPD (+ / −)	16/26
Resected pulmonary lobe	
LUL	13
LLL	3
RUL	13
RML	3
RLL	9
RUL + RML	1
Macchiarini scale	
Score 1	8 (UL: 5, ML: 1, LL: 2)
Score 2	17 (UL: 13, LL: 4)
Score 3	17 (UL: 8, ML: 2, LL: 6, UML: 1)
Chest tube duration (days)	2.0 [1–31]

*COPD* chronic obstructive pulmonary disease; *LLL* left lower lobe; *LUL* left upper lobe; *RLL* right lower lobe; *RML* right middle lobe; *RUL* right upper lobe; *UL* upper lobe; *ML* middle lobe; *LL* lower lobe; *UML* upper and middle lobe

### Treatment outcome for air leaks

In all 8 patients (100%) with MSS 1 air leak, the leaks could be controlled using VSCS alone. Conversely, the leaks in only 9 patients (52.9%) with MSS 2 air leak and 6 patients (35.3%) with MSS 3 air leak were controlled using VSCS alone (Table 2).

**Table 2:** Number of patients treated with VIO soft coagulation system classified using the Macchiarini scale score

MSS	VIO soft coagulation system alone (N = 23)	Additional procedures (N = 19)	*p*-value
1	8 (100%)	0	0.01
2	9 (52.9%)	8	
3	6 (35.3%)	11	

*MSS* Macchiarini scale score

**Fig. 3:**
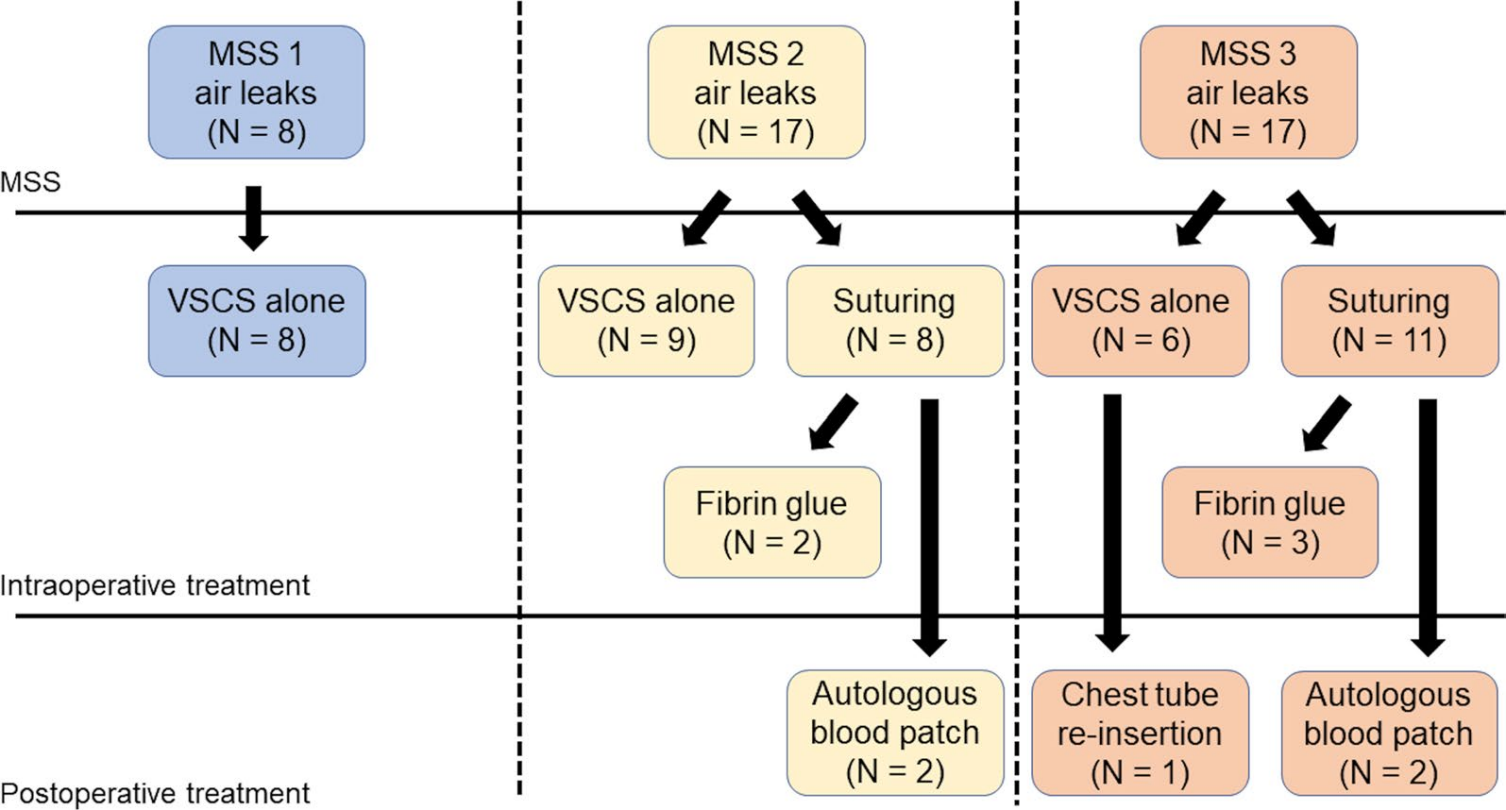
Treatments for pulmonary fistula classified using the Macchiarini scale score. *MSS* Macchiarini scale score; *VSCS* VIO soft coagulation system

Among the 8 patients with MSS 1 air leak controlled using VSCS alone, no patient had delayed pneumothorax requiring intervention. However, 1 patient with MSS 3 air leak controlled using VSCS developed pneumothorax and required chest tube insertion 2 months after lobectomy (Fig. 3). All patients with MSS 2 and 3 air leaks that remained uncontrolled following VSCS underwent suturing as an additional procedure for pulmonary fistula. Fibrin glue was used in 2 patients with MSS 2 air leak and 3 patients with MSS 3 air leak that were uncontrolled following VSCS (Fig. 3). Two patients with MSS 2 air leak and 2 patients with MSS 3 air leak underwent suturing and required autologous blood patch for prolonged air leaks postoperatively (Fig. 3).

## Discussion

VSCS is an electrosurgical output device newly developed in Germany. The main feature of VSCS, which is used primarily for arresting hemorrhage, is autoregulation of the output voltage by a built-in computer that maintains the temperature below the boiling point without generating sparks and causing carbonization of tissues [8–10]. The use of the VSCS for hemostasis was introduced at our institution in 2010. Subsequently, it was used for air leak treatment during pulmonary resection because it was occasionally observed that air leaks from lung injury sites were controlled when these sites were coagulated with the VSCS to arrest hemorrhage. The Watt/Effect settings of VSCS were tested while arresting hemorrhage from the chest wall, in order to decide the appropriate settings. Under these conditions, the VSCS at 80 Watt/Effect 5 is used to treat minor air leaks during pulmonary resection because it provides adequate pressure resistance without pulmonary artery injury. In a previous study, we also reported that the VSCS at 80 Watt/Effect 5 was the most appropriate setting for air leak treatment without causing surrounding tissue injury in a mouse pulmonary air leak model [13]. Soft coagulation with the VSCS is a quick and easy procedure that can be effective for minor lung air leaks due to injuries such as pinholes caused by lung stapling and small defects of the pleura and/or lung parenchyma caused by node dissection.

In some reports on air leak treatments, air leaks were graded using the MSS [2, 14, 15]. Filosso et al. reported that stapling and suturing were performed for controlling MSS 3 air leaks, and human fibrinogen-thrombin patches were effective for controlling MSS 1 and 2 air leaks [2]. The most popular surgical sealant to treat lung air leakages in Japan is fibrin glue [5]. Although these procedures are useful for treating air leaks, all of them involve artificial materials, and consequently, foreign body reactions may occur. Procedures with artificial materials are avoided in young patients due to intrapleural adhesions that may occur, rendering the second ipsilateral thoracic surgery difficult. Furthermore, these procedures are expensive, and we limit their multiple applications in a single patient. In contrast, soft coagulation with the VSCS can be used repeatedly for multiple lung air leaks without any artificial materials. Therefore, it can reduce the use of artificial materials for air leak treatment and is associated with lower healthcare costs.

In this study, MSS 1 air leaks could be well controlled using VSCS alone. However, 47.1% and 64.7% of patients with MSS 2 and 3 air leaks, respectively, had uncontrolled air leaks following treatment with VSCS alone, and additional procedures were needed during pulmonary resection. We reported that the fibrin membrane and fibrin clots may be involved in the recovery from air leaks with the VSCS [13]. Although deficiencies of capillary blood vessels and fibrin due to emphysema and/or the area and depth of the lung injury site may cause uncontrolled air leaks following treatment with VSCS alone, additional research is required to confirm the factors underlying uncontrolled air leaks.

No patient with MSS 1 air leak controlled using VSCS alone had delayed pneumothorax requiring intervention in our study cohort. In our institution, the chest tube without air leak is removed on the first postoperative day after lobectomy to prevent pain and postoperative pneumonia. Soft coagulation with the VSCS can shorten the chest tube duration of the patients with intraoperative minor air leak, and may be useful for preventing postoperative complications.

Our study has several limitations. First, this was a retrospective study. Second, lung conditions were not even across the study population. Third, there was no comparative group with which patients that had their air leak controlled using VSCS could be compared with, because the VSCS was always used for air leak treatment during this period. Finally, this study included a relatively small population. Therefore, additional studies are required to confirm VSCS indications for air leaks.

## Conclusions

In conclusion, MSS 1 air leaks from the interlobar lung parenchyma could be well controlled with treatment using VSCS. Therefore, soft coagulation with the VSCS could serve as an alternative method for treating air leaks during pulmonary resection surgery.

## Data Availability

The datasets used and/or analyzed during the current study are available from the corresponding author on reasonable request.

## Abbreviations

VSCS: VIO soft coagulation system
VATS: Video-assisted thoracoscopic surgery
MSS: Macchiarini scale score
